# Assessing executive functioning in higher education: development and structural validation of a new self-report scale

**DOI:** 10.3389/fpsyg.2025.1613290

**Published:** 2025-06-26

**Authors:** Samuel Zamora-Lugo, Vicenta Reynoso-Alcántara, Javier Sanchez-Lopez, Samana Vergara-Lope, Elizabeth Ocampo-Gómez, María Luisa García-Gomar, Cynthia Torres-González, Gloria Nélida Avecilla-Ramírez, Cintli Carolina Carbajal-Valenzuela, Melissa Calderón, Almitra Vázquez-Moreno, Rubén Flores-González, Carlos César Contreras-Ibáñez, Félix Ángel Montero-Domínguez, Aurora de Jesús Mejía-Castillo, Alicia Abundis-Gutierrez, José Elías Sánchez-Cid, María Iliana Osorio-Guzmán, Gregorio García-Aguilar, Agustín Jaime Negrete-Cortes, Naghelli Cisneros Báez, Luz María Martell Ruiz, Paulina Campos Romero, Rossana de Fátima Cuevas-Ferrera

**Affiliations:** ^1^Facultad de Psicología Campus Xalapa, Universidad Veracruzana, Xalapa, Veracruz, Mexico; ^2^Escuela Nacional de Estudios Superiores Unidad Juriquilla, Universidad Nacional Autónoma de México, Querétaro, Mexico; ^3^Instituto de Investigaciones en Educación, Universidad Veracruzana, Xalapa, Veracruz, Mexico; ^4^Facultad de Ciencias de la Salud, Universidad Autónoma de Baja California, Tijuana, Baja California, Mexico; ^5^Facultad de Ciencias Administrativas, Sociales e Ingeniería, Universidad Autónoma de Baja California, Mexicali, Baja California, Mexico; ^6^Facultad de Psicología y Educación, Universidad Autónoma de Querétaro, Querétaro, Mexico; ^7^Laboratorio de Psicología Experimental, Universidad Autónoma del Estado de Hidalgo, Atotonilco de Tula, Hidalgo, Mexico; ^8^Centro de Estudios de Opinión y Análisis, Universidad Veracruzana, Xalapa, Veracruz, Mexico; ^9^Departamento de Sociología, Universidad Autónoma Metropolitana-Iztapalapa, Ciudad de México, Mexico; ^10^Centro de Investigación en Compartiendo y Salud, Universidad de Guadalajara, Guadalajara, Jalisco, Mexico; ^11^Facultad de Psicología, Benemérita Universidad Autónoma de Puebla, Puebla, Mexico; ^12^Facultad de Psicología, Universidad Autónoma de Tlaxcala, Tlaxcala, Mexico; ^13^Facultad de Psicología, Universidad Autónoma de Yucatán, Mérida, Yucatán, Mexico

**Keywords:** executive functions, higher education, university students, scale development, psychometric validation, ecological validity, self-perception of executive functioning

## Abstract

**Introduction:**

Executive functions are essential cognitive processes that support goal-directed behavior, self-regulation, and academic performance in higher education. However, few assessment tools provide psychometrically validated and contextually appropriate measures for university populations.

**Methods:**

This study presents the development and structural validation of the Executive Functions Scale in Higher Education (EFEES), a self-report instrument designed to evaluate university students’ self-perceived executive functioning. The scale was developed through a theory-driven approach that defined 10 core dimensions, validated behavioral indicators with expert input, and generated positively framed items tailored to the academic context. A total of 1,538 undergraduate students from 12 Mexican universities (*M* = 20.6, SD = 1.69) completed the instrument.

**Results:**

Exploratory and confirmatory factor analyses supported a four-factor structure—organization, self-control, attentional and inhibitory control, and planning and time management—accounting for 38% of the variance. The scale demonstrated high internal consistency across factors (Cronbach’s *α* = 0.84–0.97; McDonald’s *ω* = 0.84–0.99).

**Discussion:**

Findings confirm the structural validity and reliability of the EFEES and highlight its utility in identifying executive-function profiles associated with students’ cognitive and academic development. Although initially validated in a Mexican sample, the EFEES was conceptually designed for cross-cultural applicability and can be adapted to diverse higher education contexts. The scale offers a theoretically grounded, psychometrically sound, and practically relevant tool for research and educational interventions aimed at supporting student success.

## 1 Introduction

Executive functions are cognitive processes responsible for guiding, directing, and regulating cognitive, emotional, and behavioral functions, particularly in novel situations requiring problem-solving (Brunnert et al., 2009). Lezak et al. (2012) define executive functions as a collection of abilities that enables individuals to engage in goal-directed, independent, and self-regulated behavior. Executive functions also encompass affective components, often referred to as “hot executive functions,” which are activated in emotionally charged or motivationally significant situations. These affective components involve the regulation of emotional responses, delay of gratification, and the capacity to make decisions based on reward, punishment, or social feedback, playing a critical role in adaptive, real-world functioning (Zelazo and Müller, 2002).

These functions are essential for adapting to changing circumstances and navigating novel or challenging contexts, where individuals must take control of their attention regulation. This allows for the conscious regulation of thoughts, actions, and emotions (Zelazo et al., 2024). Executive functions are crucial in fostering independence, as they help individuals organize their behavior over time, inhibit impulsive responses, and work toward long-term goals (Dawson and Guare, 2010). Thus, they are not only concerned with immediate actions but also involve a future-oriented perspective, including the capacity to plan, anticipate objectives, and devise strategies for achieving them. By leveraging these skills, individuals can plan and organize activities, maintain sustained attention, demonstrate perseverance, and complete tasks efficiently. Additionally, executive functions support emotional regulation and cognitive monitoring, enabling individuals to work more effectively and regulate their own behavior (Dawson and Guare, 2010). They are also fundamental for establishing and maintaining successful interpersonal relationships (Brunnert et al., 2009).

The development of executive functions is most prominent during childhood, a period marked by rapid neurological and behavioral changes, and progresses more gradually through adolescence and into early adulthood. According to Flores and Ostrosky-Shejet (2012), executive functions mature rapidly during early and middle childhood, with many reaching a developmental plateau by mid adolescence. However, more complex abilities such as sequential planning and abstract reasoning, continue to develop into early adulthood. A growing body of research supports the notion that these functions can be taught and enhanced through targeted interventions at all ages (Diamond and Ling, 2020; Escolano-Pérez et al., 2022). Evidence suggests that executive-function training is effective in adulthood (Zhang et al., 2023), particularly within university settings and among students with attention deficit disorder (LaCount et al., 2018). However, achieving meaningful real-world transfer of such training remains a challenge (Zelazo et al., 2024). Nonetheless, the potential for enhancing executive functions through training highlights their relevance in educational interventions.

Executive functions are a key determinant of academic and professional success. In recent years, various studies have demonstrated the link between executive functions and academic achievement (Knouse et al., 2014; Nadinloyi et al., 2013; Stadler et al., 2016). For example, Escolano-Pérez et al. (2022) suggest that executive functions, when applied within the learning process, can be considered a form of self-regulated learning. Supporting this view, a systematic literature review conducted by Dörrenbächer-Ulrich and Bregulla (2024) found low to moderate correlations between self-regulated learning and executive functions in university students and adults. According to their analysis, these associations were stronger when assessed through self-report questionnaires rather than cognitive tests. Furthermore, researchers such as Escolano-Pérez et al. (2022) and Kamradt et al. (2021) concur that the development of executive functions is associated with academic success in higher education. Given the high dropout and failure rates in Latin American universities, studying the cognitive factors related to academic performance is critical (González-Fiegehen, 2007).

Many students enter university with underdeveloped executive functions, such as organization, emotional regulation, planning, attentional control, motivation, perseverance, and metacognition. These deficits manifest in poor-quality academic work, difficulties in time and resource management, limited attention span, ineffective academic planning, and a tendency to prioritize short-term gratification over long-term goals (Kamradt et al., 2021). Such challenges hinder students’ ability to adapt to the university environment, where autonomous learning is essential (Escolano-Pérez et al., 2022).

Additionally, executive functioning is linked to health-related behaviors in university settings. For instance, executive dysfunction has been associated with alcohol abuse (Brunelle and Flood, 2016). Marshall and Elliott (2005) report that metacognition, working memory, planning, and organization skills are significant predictors of healthy eating, emphasizing the role of executive functions in managing daily behaviors such as diet, which impact students’ overall well-being and academic performance. This aligns with findings of McGrath et al. (2023), who observed that students engaging in healthy behaviors tend to exhibit stronger executive functioning, reinforcing the importance of these cognitive processes in regulating behaviors that affect health and well-being. Similarly, Gustavson et al. (2020) analyzed the relationship between executive functions and worry tendencies in university students, concluding that executive dysfunction may predispose students to heightened anxiety, which negatively impacts both academic performance and emotional well-being. These findings highlight the broader significance of executive functions, not only for academic achievement but also for promoting healthy lifestyle habits that can prevent negative physical and mental health outcomes.

Executive functions should be prioritized in contemporary education, as the ultimate goal is to equip students with the skills necessary to become autonomous citizens in an evolving world. Therefore, fostering their development should be an educational priority (Zelazo et al., 2024) and be systematically integrated into university curricula (Escolano-Pérez et al., 2022).

Despite the well-established relevance of executive functions in higher education, few instruments demonstrate contextual validity within this setting. Accurate evaluation of executive functioning is essential not only to identify students at risk of academic underperformance but also to guide the design of interventions that foster autonomy, self-regulation, and adaptive learning strategies, skills critical for success in university environments (Zelazo et al., 2024; Escolano-Pérez et al., 2022).

Executive functions can be evaluated using performance-based tests (direct measures) or self-report scales (indirect measures), each with distinct advantages and limitations. Performance-based assessments, such as the Neuropsychological Battery of Executive Functions and Frontal Lobes *Batería Neuropsicológica de Funciones Ejecutivas y Lóbulos Frontales-2* [BANFE-2] – (Flores et al., 2014), provide standardized and precise measurements but require individual administration, specialized expertise, and significant resources, making them impractical for widespread use outside clinical settings. Additionally, some scholars, such as Barkley (2012), have raised concerns regarding the ecological validity of these assessments, as they are conducted in controlled environments that do not necessarily reflect everyday academic demands. In contrast, self-report scales offer a more accessible, cost-effective, and scalable approach; however, they rely on the subjective perception of respondents. One commonly used tool is the Behavior Rating Inventory of Executive Function-Adults Version (BRIEF-A; Roth et al., 2005), which, despite its utility in educational contexts, is not specifically tailored for university settings. Furthermore, many commercially available self-report instruments are expensive, difficult to acquire, and lack validation for the educational context. This distinction between assessment types is particularly relevant in higher education, where executive function demands unfold in real-life academic contexts that are difficult to replicate in controlled environments. While performance-based tests provide objective indicators, they may not fully capture how students manage attention, emotion, and behavior in everyday academic life. Self-report instruments, despite their subjectivity, may offer greater ecological validity by reflecting students’ actual experiences and challenges in the university setting (Follmer, 2021). This underscores the need for accessible, reliable, and ecologically valid tools specifically designed to assess executive functions in higher education populations.

Given these challenges, a growing need arises to develop and validate assessment tools specifically designed for evaluating executive functions in university students. Several initiatives have been undertaken in different contexts to address this need. For instance, Kamradt et al. (2021) validated the Barkley Deficits in Executive Functioning Scale for a sample of American university students. Similarly, Wallace and Hoskyn (2022) designed and evaluated the Strategy Awareness and Use Questionnaire, which measures students’ self-reported use of cognitive, socio-emotional, and behavioral strategies to regulate attention in academic settings. Additionally, Strait et al. (2020) examined the psychometric properties of a revised version of the Executive Skills Questionnaire developed by Dawson and Guare (2010), which assesses executive functions relevant to academic performance in higher education.

Recently, efforts have been made to develop and adapt executive-function assessment tools for Spanish-speaking populations. Escolano-Pérez et al. (2022) conducted a cross-cultural adaptation of the Amsterdam Executive Function Inventory for Spanish university students, measuring attention, self-control, and planning using a brief 10-item scale. This adaptation followed standardized procedures for cross-cultural validation, including translation, back translation, and psychometric analyses, resulting in a tool with good internal consistency and factorial invariance across gender and academic disciplines. However, the study acknowledged limitations such as the inability to assess convergent validity due to the lack of comparable instruments in Spanish and a relatively small, non-representative sample from a single university.

In contrast, Ramos-Galarza et al. (2023) developed a 31-item scale to assess executive functions in university students from Ecuador and Chile, including dimensions such as conscious monitoring of responsibilities, supervisory attentional systems, behavioral self-regulation, cognitive verification for learning, emotional regulation, and problem-solving strategies. While their study represents a significant contribution to the field and offers preliminary evidence of reliability and factorial validity, certain aspects of the scale’s conceptual and methodological design remain open to further development. For instance, the relationship between theoretical definitions and behavioral indicators could be more explicitly articulated to strengthen dimensional clarity and avoid potential overlap. Additionally, limited information is provided on the rationale guiding item construction. These observations underscore the ongoing need for theoretically grounded instruments that ensure both conceptual coherence and contextual sensitivity in university settings.

In the present study, we proposed a design methodology guided by a process that emphasizes conceptual clarity and contextual relevance. This process includes defining each dimension based on an extensive theoretical review, carefully aligning behavioral indicators with these definitions, and incorporating expert judgment to ensure precision in item construction. Moreover, this study followed rigorous psychometric methods, including expert validation and iterative refinement of items. These steps improve on previous tools by ensuring greater conceptual clarity and validity in higher education contexts.

Developing a valid and reliable scale to assess executive functions in the higher education context can provide valuable insight into how students perceive and manage their cognitive and behavioral regulation in academic settings. In the medium and long term, such a tool may contribute to identifying general patterns of executive functioning and offer preliminary data to guide the design of targeted interventions aimed at strengthening these abilities. However, given the complexity of executive functions, such insights are best interpreted in conjunction with complementary methodologies, such as behavioral observation, academic performance metrics, or experimental tasks, to ensure a comprehensive understanding of student needs.

Furthermore, assessing executive-function levels can enrich the interpretation of academic performance, particularly in cases where learning difficulties are not explained by intellectual or emotional impairments. Students with underdeveloped executive functions may face challenges in effectively engaging with learning tasks, including difficulties with planning, organization, sustained attention, and time management. Since executive functions are amenable to improvement through appropriate interventions, assessment plays a key role in identifying areas of support and informing the design of strategies to enhance these skills. These interventions could positively influence not only academic performance but also broader aspects of students’ wellbeing (Escolano-Pérez et al., 2022; Kamradt et al., 2021).

Thus, the objective of this study was to design a self-report scale grounded in contemporary executive-function theories to assess the self-perceived executive functioning of higher education students and validate in Mexican population. Executive functions were conceptualized as a set of higher-order processes that support goal directed behavior, self-regulation, and adaptive functioning in complex academic contexts. This study draws upon contemporary approaches that integrate cognitive, emotional, and behavioral components of executive functioning, with an emphasis on their application in real-life educational environments. Rather than relying on a single theoretical model, we conducted an extensive review of literature on executive-function assessment and its application in educational settings, which served as the basis for defining and operationalizing the dimensions of the construct. These definitions informed the development of the behavioral indicators included in the scale (see Appendix 1). Our scale was conceptually designed to address challenges common to university students in broader academic contexts, although the present validation was conducted within the Mexican higher education system.

## 2 Materials and methods

### 2.1 Item development phase

This study employed a cross-sectional observational design, which allows for data collection at a single point in time without manipulating the variables under study. This approach is commonly used in psychometric research to evaluate measurement properties such as construct validity and internal consistency in a naturalistic setting. As noted by Arafat (2016), although validation studies often adopt a cross-sectional structure, they involve a distinct set of methodological procedures, including conceptual definition, item development, expert evaluation, and statistical validation, that set them apart from standard observational research. Therefore, the selected design is appropriate for the study’s objectives and consistent with established practices in instrument development.

The Executive Functions Scale in Higher Education (EFEES, from its Spanish acronym *Escala de Funciones Ejecutivas en Educación Superior*) was developed to measure and quantify university students’ self-perception of their executive functions. This instrument uses Likert-type items through which students evaluate their own executive functioning capabilities.

A distinctive feature of this instrument is its positive framing of executive function attributes. Instead of focusing on deficits, the items describe characteristics associated with strong executive functioning. While this approach is shared by some recent instruments (e.g., Ramos-Galarza et al., 2023), the present scale is grounded in a theory-based construction process that emphasizes conceptual precision and ecological relevance. Executive function components are assessed as adaptive traits observable in everyday academic settings, defined through clear behavioral indicators aligned with the educational context. Thus, the scale offers a structured, operational framework for identifying strengths and targets for intervention in university students.

Following the guidelines of Boateng et al. (2018) and Naglieri and Goldstein (2014), special attention was given to the conceptual definitions that served as the foundation for the behavioral indicators included in the scale. To ensure content validity, the definitions and indicators were developed based on an extensive review of literature on executive-function assessment (Dawson and Guare, 2010; Diamond, 2013; Greenstone, 2011; Kennedy, 2017; McCloskey et al., 2008; Meltzer, 2010; Naglieri and Goldstein, 2014; Najdowski, 2017; Roth et al., 2005) and their contextualization within the educational domain (see Appendix 1). The scale encompasses the following dimensions: cognitive flexibility, inhibitory control, working memory, attentional control, emotional control, task initiation, planning, organization, time management, and self-monitoring.

To ensure the conceptual validity of the proposed dimensions, a panel of six expert judges reviewed and provided feedback on the definitions and corresponding behavioral indicators. All experts held doctoral degrees in neuroscience and had specific training in the assessment of executive functions and the development of psychological instruments. Their evaluation focused on the clarity, coherence, and theoretical consistency of each definition and its alignment with the targeted executive function. They also reviewed whether each behavioral indicator appropriately reflected the construct it intended to represent. The experts provided written feedback and suggestions, which were used to refine and adjust the definitions and indicators prior to item construction. Although this phase did not involve a formal quantitative validation, the expert review contributed to ensuring the theoretical soundness and content relevance of the instrument.

Based on these validated behavioral indicators, 122 items were drafted, ensuring that at least one item corresponded to each behavioral indicator. Each item consisted of two components: a statement describing the behavior and an example contextualized for the educational setting. For instance, Item 202 states: “I can voluntarily ignore my thoughts or emotions.” Example: “*You intentionally stop paying attention to unrelated thoughts or emotions and focus on what the professor is saying in class.”*

### 2.2 Item validation phase

The preliminary pool of 122 items underwent expert review by a panel of 13 judges. All reviewers were psychologists with expertise in executive functions, psychological assessment, or both. Among them, eight held doctoral degrees (five of which were in neuroscience), two held master’s degrees, and three held bachelor’s degrees in psychology. Although the experts’ exact years of professional experience were not formally documented, their levels of expertise were estimated based on self-reported experience, thus, five judges reported extensive experience in executive function assessment, five had moderate experience, and three had limited experience. Regarding expertise in psychological instrument development, seven had extensive experience and six had moderate experience. Notably, five of these judges had also participated in the earlier review of the theoretical definitions and behavioral indicators, ensuring continuity and conceptual alignment throughout the scale’s development.

Each evaluator received an email containing a spreadsheet link with the scale’s instructions, dimensions, behavioral indicators, and items, along with an assessment form comprising six dichotomous (Yes/No) questions: (1) Does the item correspond to the intended dimension? (2) Does the item align with the behavioral descriptor? (3) Is the item relevant for executive function assessment? (4) Is the item clearly formulated? (5) Is the item’s language appropriate for university students? (6) Is the response scale suitable? The form also included a comments section for specific suggestions.

Each question targeted a distinct psychometric or contextual attribute of the item. Question 1 assessed the item’s alignment with the theoretical construct; Question 2 evaluated its consistency with the behavioral descriptor; Question 3 examined its relevance for assessing executive functions; Question 4 addressed syntactic and semantic clarity; Question 5 considered linguistic and contextual appropriateness for the target population; and Question 6 evaluated the adequacy of the response format in capturing variability in the construct.

Items were eliminated if two or more experts answered “No” to questions 1, 2, or 3, as these reflected foundational issues regarding construct validity. A total of 16 items were discarded for this reason. For example, the item *“… respeto los espacios de trabajo, por ejemplo, me quedo en mi lugar.* [… I respect shared workspaces, for example, I stay in my place.]” was originally intended to reflect the dimension “Thinking before acting, controls impulsive behaviors,” but was deemed misaligned with the construct.

A further 29 items were revised based on expert feedback to questions 4, 5, or 6, which referred to wording clarity, appropriateness of language, or response scale suitability. For instance, the item *“… controlo la atención que le presto a los estímulos del entorno, decidiendo a qué presto atención y qué cosas ignoro, por ejemplo, me enfoco en escuchar lo que dice el maestro y evito prestar atención a los ruidos del ambiente* [I control the attention I give to environmental stimuli, deciding what I focus on and what I ignore, for example, I concentrate on listening to what the teacher says and avoid paying attention to background noise.]” was reformulated as “*Controlo a qué presto atención y qué cosas ignoro. Te enfocas en escuchar lo que dice el maestro y evitas prestar atención a los ruidos del ambiente.* [I control what I pay attention to and what things I ignore. You focus on listening to what the teacher is saying and avoid paying attention to ambient noises.].”

Based on general feedback, items were restructured to clearly separate the core statement (in first person) from the illustrative example (in second person), enhancing readability and contextual relevance.

Following this expert review process, 106 items were retained and refined as necessary. Additionally, three verification items were included to detect inattentive or careless responses (e.g., “I know how to read texts in Spanish,” where the expected response would be “Describes me perfectly”), bringing the total number of items in the scale to 109. The finalized instrument was then digitized, with all items presented in a Likert-type format, ranged from 0 (Does not describe me at all) to 5 (Totally describes me).

The revised scale was then prepared for pilot testing with university students.

### 2.3 Pilot implementation phase

Ten undergraduate psychology students from one of the participating universities took part in the pilot test. All of them met the inclusion criteria defined for the final study sample. Participants independently completed the final version of the scale on their personal mobile device. The research team recruited participants through direct contact and obtained informed consent before administering the instrument. After confirming that responses were complete, the team conducted brief individual interviews to gather qualitative feedback on the clarity, relevance, and usability of the items. This feedback did not lead to any substantial modifications to the instrument.

### 2.4 Validation phase

The final 109-item scale was administered online using the LimeSurvey Professional® platform. The survey consisted of three sections:

1. Informed Consent: Briefly explaining executive functions, study objectives, participation requirements, confidentiality provisions, potential risks, and benefits.
2. General and Sociodemographic Data: Collecting data on age, gender, state of residence, employment status, and socioeconomic status (assessed using the AMAI classification system developed by the Mexican Association of Market Intelligence and Opinion Agencies, 2022). As this classification is specific to the Mexican context, a link to the official methodological document has been provided in the References section for international readers. Participants were also asked about any neurological or psychiatric diagnoses affecting daily functioning. Academic information was collected, including the university name, field of study, and number of completed academic terms.
3. EFEES Scale: The instrument consisted of 109 items, 106 of which assessed executive functions and 3 served as verification items designed to detect inattentive or careless responding. The estimated completion time was 20 to 30 min.

Before beginning the scale, participants were shown the following instructions:

*“Below are some statements. Please assess how well each statement describes you in relation to your academic work over the past 6 months (*e.g.*, studying, attending classes, completing assignments, and working with classmates). There are no right or wrong answers; please respond honestly in a way that best reflects your experiences.”*

Response options ranged from 0 (Does not describe me at all) to 5 (Totally describes me). The survey was available between March 15 to May 22, 2022. As an incentive, participants had the option to enter a raffle for a $2000 MXN gift card from an online retailer.

### 2.5 Participants and sampling

Researchers from 12 universities across Mexico assisted in disseminating the study via social media and institutional channels: *Universidad Autónoma de Baja California*, *Universidad Autónoma de Tlaxcala*, *Universidad Autónoma de Querétaro*, *Universidad Autónoma Metropolitana*, *Benemérita Universidad Autónoma de Puebla*, *Universidad Nacional Autónoma de México*, *Universidad Autónoma del Estado de Hidalgo*, *Universidad Autónoma de Yucatán*, *Universidad de Guadalajara*, *Instituto Tecnológico de Sonora*, *Universidad Iberoamericana* y *Universidad Veracruzana*.

A convenience sampling method was used, inviting all undergraduate students to participate.

#### 2.5.1 Inclusion criteria

Age 18–25 years.

Currently enrolled in a Mexican university undergraduate program.

#### 2.5.2 Exclusion criteria

Declining to provide informed consent.

#### 2.5.3 Elimination criteria

- Not completing the scale.
- Completing the survey in under 10 min.
- Having been enrolled in their program for more than 5 years.
- Incorrectly answering one or more of the three verification items.

The EFEES was completed by 3,791 participants, of whom 1,538 met the inclusion criteria and provided valid responses. Of the excluded participants, 1,615 left items unanswered, 28 completed the survey too quickly, 67 were outside the age range, 12 had exceeded 5 years of enrollment or had graduated, and 531 failed one or more verification items.

### 2.6 Ethical considerations

This research adhered to the ethical principles outlined in the Declaration of Helsinki (World Medical Association, 2013) for studies involving human participants. Prior to participation, individuals provided informed consent, which included a clear explanation of the study’s objectives, procedures, and potential benefits. Participants were informed of their right to withdraw at any time without facing any consequences. Confidentiality was strictly maintained, ensuring that all data and results remained anonymous, and participants’ identities were fully protected (Pan American Health Organization and Council for International Organizations of Medical Sciences, 2017).

The study received approval from the Research Ethics Committee (REC) and was officially registered with the National Bioethics Commission of Mexico (CONBIOETICA-30-CIE-006-20191210).

### 2.7 Statistical analysis

For the statistical analysis, RStudio (RStudio Team, 2020) was used, drawing on data from the 1,538 participants. Since the survey platform required responses to all items, no missing values were present in the dataset analyzed. Cases in which participants exited the survey without completing all items were excluded from the analysis. Initially, a descriptive analysis was conducted on the general and sociodemographic data, using measures of central tendency, dispersion, frequency, and percentage distributions.

For the structural analysis, item selection was refined by calculating the item-total correlation using the *item.total* function from the *multilevel* package (Bliese, 2022). Items with values ≥ 0.4 were retained (Zijlmans et al., 2018). Subsequently, an exploratory factor analysis (EFA) was performed using a polychoric correlation matrix (*hetcor* function, *polycor* package; Fox, 2022). Factorization adequacy was confirmed through Bartlett’s test Bartlett (1950) of sphericity and the Kaiser-Meyer-Olkin (KMO) measure. The optimal number of factors was determined via parallel analysis, applying the *fa.parallel* and *n_factors* functions. Factor extraction was conducted using the weighted least squares method with Promax rotation, executed through the *fa* function of the *psych* package (Revelle, 2024).

Following this, a confirmatory factor analysis (CFA) was conducted using the *cfa* function from the *lavaan* package (Rosseel, 2012). Model fit was assessed using goodness-of-fit indices, including χ^2^ with degrees of freedom, Comparative Fit Index (CFI), and Tucker-Lewis Index (TLI), where values ≥ 0.90 were considered acceptable (Schumacker and Lomax, 2016). Additionally, the root mean square error of approximation (RMSEA) and the Standardized Root Mean Square Error (SRMR) were evaluated; values in the range of 0.50 to 0.80 and values less than 0.08, respectively, were considered satisfactory (Chen et al., 2008).

The same sample was used for both the EFA and CFA. This decision was supported by bootstrapping and K-fold cross-validation procedures. This statistical technique allows estimating the precision of a parameter by generating multiple samples from the original data, including observations with replacement. In this case, using 1,000 iterations in Bootstrapping and 5 folds, similar parameters were observed to those presented in the EFA and CFA with the same sample. Finally, internal consistency was assessed using Cronbach’s alpha coefficient (Cronbach, 1951) and McDonald’s Omega coefficient, both calculated using the *alpha* function from the *psych* package. Reliability values exceeding 0.80 were considered indicative of satisfactory internal consistency (Viladrich et al., 2017).

## 3 Results

The mean age of participants was 20.6 years (*SD ± 1.69*), representing 24 different states across the Mexican Republic, predominantly Veracruz, Puebla, Mexico City, and the State of Mexico. Most participants reported not being employed, and 62.5% were classified within stratum C, corresponding to a middle-class socioeconomic level.

Students were enrolled in various universities across Mexico, with the *Universidad Veracruzana*, *Universidad Autónoma Metropolitana*, *Benemérita Universidad Autónoma de Puebla*, and *Universidad Autónoma de Baja California* being the most represented institutions. Participants came from 145 different undergraduate programs, with the Health Sciences area being the most prevalent. Most of these programs followed a semester-based system. The average duration of students’ enrollment in their programs was 2.1 years (*SD* ± *1.28*). Table 1 presents the full sociodemographic details.

**Table 1:** Sociodemographic characteristics of the participants.

	Frequency	Percentage
Age		
18	166	10.8
19	283	18.4
20	310	20.2
21	307	20
22	236	15.4
23	149	9.7
24	72	4.7
25	14	0.9
Sex		
Female	1,028	66.9
Male	489	31.8
Other or I prefer not to answer	20	1.3
State of residence		
Veracruz	404	26.3
Puebla	266	17.3
Mexico City	194	12.6
State of Mexico	141	9.2
Baja California	108	7
Querétaro	108	7
Tlaxcala	96	6.2
Yucatan	58	3.8
Hidalgo	47	3.1
Rest of the Country	115	7.5
Employment status		
Unemployed	1,175	76.4
Part-time employed	313	20.4
Full-time employed	50	3.3
Socioeconomic level		
A/B	262	17
C+	357	23.2
C	324	21.1
C-	280	18.2
D+	205	13.3
D	105	6.8
E	5	3
Diagnosis received		
Has been diagnosed	88	5.7
Has not been diagnosed	1,272	82.7
Not sure	178	11.6
University		
*Universidad Autónoma de Baja California*	110	7.2
*Universidad Autónoma de Tlaxcala*	90	5.9
*Universidad Autónoma de Querétaro*	82	5.3
*Universidad Autónoma Metropolitana*	305	19.8
*Benemérita Universidad Autónoma de Puebla*	299	19.5
*Universidad Nacional Autónoma de México*	57	3.7
*Universidad Autónoma del Estado de Hidalgo*	52	3.4
*Universidad Autónoma de Yucatán*	56	3.6
*Universidad Veracruzana*	403	26.2
Other	83	5.4
Program area		
Administrative economic	189	12.3
Exact Sciences	268	17.4
Health Sciences	684	44.5
Social or human sciences	284	18.5
Arts	43	2.8
Life sciences	69	4.5
Other	1	0.1
Program type		
Semiannual	1,221	79.4
Quarterly	307	20
Other	10	0.7

Socioeconomic level was assessed using the AMAI (Mexican Association of Market Intelligence and Opinion Agencies, 2022) classification, which categorizes individuals as follows: A/B (202 points or more), C+ (168–201 points), C (141–167 points), C– (116–140 points), D+ (95–115 points), D (48–94 points), and E (0–47 points). Diagnosis received refers to whether participants have been diagnosed within the past year with any neurological or psychiatric condition that affects their daily functioning.

### 3.1 Detection of the factor structure of the executive functions scale in higher education

The structural analysis was conducted in three stages. First, item reduction was performed using item-total correlation analysis. Next, an EFA was conducted to determine a preliminary factor structure. Finally, a CFA was conducted to validate the proposed structure.

Items were reduced using adjusted item-total correlations, which measure each item’s relationship with the total score (excluding the item being analyzed). From the original 106 items (not including the three validity check items), eight items with coefficients below 0.40 were eliminated, resulting in a final set of 98 items (see Appendix 2). The suitability of these 98 items for factorization was assessed using a polychoric correlation matrix, along with the KMO test (*KMO = 0.98*) and Bartlett’s test of sphericity (*χ^2^* = 73613.94, *df* = 4,753, *p* < 0.05), both of which confirmed that the correlation matrix was appropriate for factor analysis (López-Aguado and Gutiérrez-Provecho, 2019).

**Figure 1:**
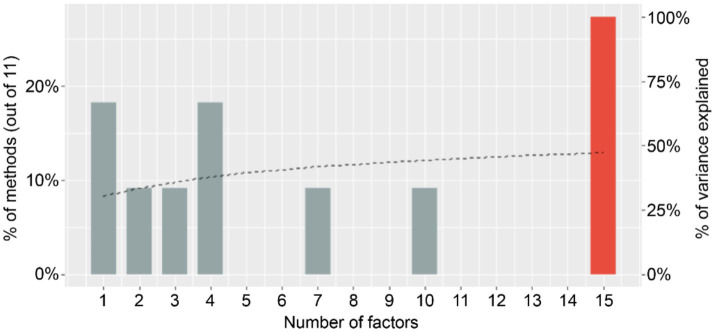
Factor extraction. The black dotted line represents the percentage of variance explained for each number of factors simulated through parallel analysis.

To determine the optimal number of factors, multiple extraction methods were evaluated using the *n_factors function* from the parameters package, initially suggesting 15 factors (see Figure 1). However, considering theoretical perspectives and the recommendation to select the simplest model, a four-factor solution was adopted, accounting for 38% of the variance (Lüdecke et al., 2020).

Factor extraction was conducted using the *weighted least squares* method with *Promax* rotation. Of the 98 items, 31 were eliminated for failing to meet established criteria: (1) factor loading below 0.40, or (2) cross-loadings of 0.35 or higher on other factors. The final instrument consisted of 67 items (plus three validity check items), grouped into four factors: organization (F1) with 28 items, self-control (F2) with 14 items, attentional and inhibitory control (F3) with 15 items, and time planning and management (F4) with 10 items (see Appendix 3).

The initial EFA suggested a 4-factor structure. This structure was evaluated using CFA in the same sample, showing an acceptable fit (*χ^2^* = 12772.5, *df* = 2,138, *p* < 0.01, *CFI* = 0.98, *TLI* = 0.98, *CFI* = 0.92, *RMSEA* = 0.057, *with a 90% confidence interval of 0.056 to 0.058, SRMR* = 0.051). To mitigate the risk of overfitting, it was validated with bootstrapping (*95% CI for loadings*) and k-Fold CV (a*verage CFI* = 0.90). The results support the proposed structure, but replication in independent samples is recommended. The standardized factor loadings ranged between 0.5 and 0.8 (*p* *<* 0.001), indicating a strong representation of each item within its assigned factor (see Appendix 3). Although modification indices suggested the addition of correlations between measurement errors, this did not lead to a significant improvement in model fit. Thus, the original model was retained, supporting the validity and internal consistency of the theoretical framework established in the EFA.

For reliability assessment, both Cronbach’s alpha and McDonald’s omega coefficients were calculated for categorical data. These complementary measures were reported because the EFA factor loadings were high (0.40 to 0.86), and no substantial differences in internal consistency estimates were anticipated. Across all factors, alpha and omega values ranged between 0.84 and 0.96, demonstrating strong internal consistency (Table 2).

**Table 2:** Internal consistency analysis of the factors using Cronbach’s alpha coefficient and the Omega coefficient adjusted for categorical data.

Dimension	No. of items	Mean (SD)	Alpha	Omega ωu-cat
F1-Organization	28	97.8 (22.2)	0.94	0.96
F2-Autocontrol	14	25.5 (10.6)	0.87	0.90
F3-Attentional control	15	45.5 (14.4)	0.92	0.93
F4-Planning	10	40.0 (7.4)	0.84	0.84
Total	67	235.87 (47.7)	0.97	0.99

### 3.2 Final structure of the executive functions scale in higher education

Although the initial development of the items was based on operational definitions of 10 executive functions widely recognized in the literature and particularly relevant in higher education, the statistical analyses did not support a structure with these 10 distinct dimensions. Instead, the findings provide strong evidence for a four-factor structure, comprising self-control, attentional control, organization, and time planning and management. While the original 10-dimensional framework offered a comprehensive and detailed perspective on executive functions, grounded in classical theoretical constructs, EFA indicated significant correlations between certain dimensions, suggesting the presence of broader latent factors. This reorganization into four more generalized factors enhances both the theoretical soundness and empirical validity of the instrument (Reise et al., 2010a; Reise et al., 2010b). By consolidating related dimensions into more cohesive and robust factors, the revised structure simplifies the scale while improving its structural validity, making interpretation of results more intuitive and enhancing its practical application in higher education contexts. The final version of the EFEES, in its Spanish version, is provided in Appendix 4.

## 4 Discussion

The results of this study provide empirical support for the structural validity of the EFEES and offer insights into how executive functions cluster into broader dimensions within the higher education context.

Building on these findings, the primary objectives of this research were to design a scale to assess executive functions in university students and validate its structural composition within the Mexican academic setting. The scale was developed to measure students’ self-perception of their executive functioning, offering a contextually grounded, theoretically robust, and psychometrically transparent alternative to existing instruments. This addresses the need for tools that are culturally adapted and methodologically explicit, enhancing the validity and applicability of executive function assessments in higher education.

The design phase of the EFEES followed a systematic process grounded in established theoretical frameworks, resulting in a coherent and context-sensitive instrument. The operational definitions of each dimension were clearly articulated and translated into behavioral indicators relevant to academic performance in higher education. The use of expert judgment during item development ensured semantic clarity and conceptual alignment, while the integration of contextualized examples contributed to the ecological validity of the scale. Furthermore, the linguistic adaptation and preliminary pilot testing reinforced the instrument’s usability and appropriateness for the target population. These design strategies provided a robust foundation for the subsequent psychometric validation.

For the validation, the findings indicate that the proposed factorial model is sufficiently robust to support the following conclusions. The initial factorial study of executive functions was based on a comprehensive analysis of subdimensions widely discussed in the literature. Although not all initially proposed subdimensions were validated, the results align with existing research that often identifies three- or four-factor structures as fundamental components of executive functions. The most commonly recognized factors include working memory, cognitive flexibility, inhibitory control, and planning (Frolli et al., 2022; Rmus et al., 2021).

To ensure the reliability of the structural analysis, several key statistical assumptions were met. Bartlett’s test confirmed that the items were significantly correlated, justifying their inclusion in a factor analysis (López-Aguado and Gutiérrez-Provecho, 2019; Stevens, 2002). The KMO test yielded a value of 0.98, indicating a highly suitable sample for factor extraction, as values above 0.70 are considered adequate (Netemeyer et al., 2003; Taherdoost et al., 2022). A polychoric correlation matrix was used, which is appropriate for ordinal polytomous data like that of the EFEES. Additionally, weighted least squares was chosen as the most suitable estimation method for such data (Lee et al., 2012; Lloret-Segura et al., 2014; Wirth and Edwards, 2007). To rotate the factors, the Promax criterion was applied, which is an oblique rotation method that allows correlation between factors. This approach is particularly relevant in psychology and the social sciences, where factors are assumingly interrelated, as opposed to orthogonal methods, which assume complete independence (Hendrickson and White, 1964).

The EFA revealed a factor structure consisting of four components: organization (F1), self-control (F2), attentional control and inhibition (F3) and planning and time management (F4). Items were retained in the final version of the scale only if they exhibited factor loadings of 0.40 or higher, a threshold widely recognized as appropriate based on the study’s sample size (Solberg et al., 2022; Spiegel et al., 2017).

The organization factor comprises items 801 through 809, which are related to the systematic use and adaptation of tools to establish order in one’s environment, materials, information, and cognitive processes. Additionally, it includes self-monitoring items—1,104, 1,105, 1,106, 1,108, 1,109, 1,111, and 1,112—which assess the individual’s capacity to observe, evaluate, and refine the strategies employed in problem-solving and task execution. Items associated with planning—701, 702, 706, 707, and 713—were also incorporated, as they address the processes of goal setting, anticipating necessary conditions for achievement, and modifying strategies when objectives are not attained. Furthermore, Item 105, which evaluates cognitive flexibility—understood as the ability to adjust strategies and thought patterns in response to changing contextual demands—was included. Items 112 and 113, which assess divergent thinking, were incorporated as they reflect the capacity to integrate diverse concepts and generate innovative solutions. In relation to working memory, Items 301, 305, and 306 were included, as they measure the ability to retain and manipulate information over short periods. Finally, Item 605, which evaluates initiative—the capacity to independently initiate tasks or projects—was also integrated into this factor. The complete list of items can be found in Appendix 2.

The organization factor represents the structured use of tools and strategies to manage information, thoughts, and materials, ultimately helping to mitigate impulsive behaviors. Research has consistently linked strong organizational skills such as effective planning, task prioritization, and maintaining an orderly study environment, to greater academic success (Barkley, 2012; Zimmerman, 2002). Additionally, organization enables the efficient allocation of time and cognitive resources, both of which are essential for completing complex tasks. In higher education, organizational skills are particularly critical for academic achievement, as students must simultaneously manage multiple projects and responsibilities. Those with well-developed organization skills can break down complex tasks into manageable steps, set realistic goals, and adapt their strategies in response to shifting academic demands (Moilanen, 2007).

The self-control factor includes Items 501, 505, and 506, which assess emotional control, specifically the ability to identify, interpret, and regulate emotions, as well as modulate their intensity and expression. Items 103 and 106, related to cognitive flexibility, were also included, as they evaluate the need to detect and modify emotional states to adapt to contextual demands. Additionally, Items 1,003 and 1,006, which focus on self-awareness, assess an individual’s ability to recognize personal strengths and weaknesses in emotional regulation, as well as identify situations that facilitate or hinder emotion management. Furthermore, Items 204, 205, 208, 209, and 211, related to inhibitory control, were incorporated, as they measure the ability to pause before acting, evaluate contextual cues, and regulate impulsive behaviors. Finally, Items 1,101 and 1,102, pertaining to self-monitoring, were integrated, assessing the ability to anticipate the outcomes of one’s actions in different situations and social interactions.

In this context, self-control is a fundamental skill for regulating emotions and behaviors, particularly in academic settings that require perseverance and stress management. Research indicates that self-control is associated with better academic performance, as it enables students to manage frustration, delay gratification, and stay committed to long-term goals (Duckworth and Seligman, 2005). The ability to inhibit impulsive behaviors and engage in thoughtful decision-making helps students minimize distractions and maintain focus on essential tasks, ultimately improving academic outcomes. In higher education, self-control is particularly crucial for adapting to new academic environments that demand greater independence and self-regulation than prior educational levels. Students who develop strong self-control skills are better equipped to manage their time and emotional resources, thereby avoiding behaviors that may hinder their academic progress (Tangney et al., 2004).

The attentional and inhibitory control factor includes Items 401, 402, 404, 405, and 406, which assess attentional control, including alertness, focused attention, selective attention, and sustained attention. Additionally, Items 302 and 303, related to working memory, were included, as they evaluate the ability to temporarily retain and manipulate information for use in subsequent tasks or problem-solving. This factor also integrates Items 201, 202, 203, 206, and 207, which assess inhibitory control, specifically the ability to voluntarily ignore distractions, suppress behavioral sequences, and regulate attention toward relevant stimuli. Furthermore, Items 110 and 111, related to cognitive flexibility, measure the ability to tolerate changes, particularly the capacity to resume interrupted routines without experiencing negative emotional reactions. Finally, Item 602, which assesses initiation, was included as it evaluates the ability to overcome procrastination.

Attentional and inhibitory control reflects an individual’s ability to maintain focus on essential tasks, sustain working memory, flexibly adapt to changes, and resist distractions by ignoring irrelevant stimuli (Diamond, 2013). Prior research suggests that sustained attention and the ability to shift focus when necessary are crucial for effective learning and complex problem-solving (Gazzaley and Nobre, 2012). In a university setting, these skills are vital for academic behaviors such as following detailed instructions, taking effective notes, and multitasking, for instance, simultaneously preparing assignments and studying for exams. Students with strong attentional control are better equipped to absorb and process information efficiently, which directly contributes to their academic success (Friedman and Miyake, 2017).

The planning and time management factor includes Items 704, 705, and 709, which assess the ability to identify the necessary steps to achieve objectives, as well as Item 710, which evaluates goal-directed persistence. Additionally, Items 901, 902, 907, and 910 related to time management, measure students’ sense of time, punctuality, and ability to prioritize tasks by dedicating more time to important or challenging activities. The factor also incorporates Item 212 from inhibitory control, which evaluates students’ ability to resist impulsive behaviors by prioritizing less immediately rewarding but more important tasks. Finally, Item 601 from initiation assesses students’ capacity to independently start their tasks or projects.

Within this dimension, planning and time management are understood as core executive functions linked to students’ ability to set goals, strategize steps to reach them, and manage available resources efficiently (Barkley, 2012). These skills are essential for developing effective study habits, meeting deadlines, and balancing academic and personal responsibilities. In higher education, where workloads are heavier and greater autonomy is required, students with strong planning and time management skills tend to perform better. They can anticipate challenges, adjust their strategies when necessary, and maintain a consistent focus on their academic goals (Claessens et al., 2007).

This study aligns with the findings of Ramos-Galarza et al. (2023), who conducted a similar study assessing seven executive functions through self-report measures in university students. Their reported internal consistency values (*α* = 0.71–0.85) and fit indices (CFI = 0.91, SRMR = 0.04, RMSEA = 0.04) were slightly lower but comparable to those of the present study. The executive functions assessed in their research included conscious monitoring of responsibilities, attentional supervisory system, conscious behavioral regulation, behavioral verification for learning, decision-making, emotional regulation, and resource management for task-solving. The present study builds on these findings by proposing a four-factor structure that provides a more comprehensive assessment of executive functions in Spanish-speaking university students.

Furthermore, the CFA indices obtained in this study were similar to those reported by Wallace and Hoskyn (2022) in their validation of the Executive Function Strategy Awareness and Use Questionnaire (SAUQ) (RMSEA = 0.05, CFI = 0.99, TLI = 0.98, χ^2^ = 587.18, df = 371, *p* = 0.001). The factors assessed by the SAUQ closely resemble those identified in the present study, as they primarily focus on executive functions related to planning, organization, and resource management. According to Wallace and Hoskyn (2022), these executive functions are distinct but interrelated components of a larger system that do not function in isolation but are crucial for effective performance across various domains, including academic achievement. Validating tools like the SAUQ and EFEES is essential for accurately assessing executive functions in university students, thereby enabling the development and implementation of more effective academic support strategies.

A notable difference between the present study and that of Wallace and Hoskyn (2022) is that the EFEES has not yet undergone concurrent validation, unlike the SAUQ, which was validated against the BRIEF-A global index and CAARS inattention/memory scale. Future research should address this limitation by conducting concurrent validation, potentially using performance-based assessments such as the Cambridge Neuropsychological Test Automated Battery (CANTAB), which evaluates various cognitive functions through computerized tasks.

The most significant contribution of this study is the development of a valid and reliable executive-function scale, freely accessible for the higher education context. While the EFEES was initially validated with Mexican university students, its design facilitates cross-cultural application in diverse higher education settings. This instrument provides researchers with a valuable tool for comparative studies across developing nations, offering potential to foster international academic partnerships and inform evidence-based policies for enhancing higher education systems.

This study contributes to the scientific community by providing a reliable and theoretically grounded instrument for assessing university students’ self-perceived executive functioning. Developed through a rigorous, theory-driven process, the EFEES integrates established executive function models, operational definitions, expert validation, and contextualized item examples to ensure semantic clarity and ecological validity. Special attention was given to linguistic and cultural relevance, particularly for Latin American students, enhancing the instrument’s applicability within higher education settings. Although initially validated in Mexico, the scale’s conceptual and methodological foundations support its future adaptation and use in diverse international contexts.

Two key characteristics of the EFEES enhance its usefulness. First, similar to the scale proposed by Ramos-Galarza et al. (2021), the EFEES defines behavioral indicators in terms of strengths rather than deficits, allowing scores to reflect areas of executive-function proficiency. Second, all items are specifically contextualized for the higher education environment, using realistic examples that clarify the role of executive functions in academic settings. This contextualization enhances the ecological validity of the scale, making it more relevant and applicable to university students’ daily experiences (Follmer, 2021).

This study successfully validated EFEES, a scale designed to measure the self-perception of executive functions in Mexican university students. The statistical analyses confirmed the suitability of the correlation matrix, supporting the extraction of a four-factor structure: self-control, attentional and inhibitory control, organization, and planning and time management. This streamlined model simplifies the original 10-dimension theoretical framework while preserving theoretical coherence, thereby facilitating the interpretation and application of the scale in educational settings. The validation of the EFEES offers a valuable tool for both academic research and practical applications, such as identifying executive-function strengths critical for academic success and designing targeted educational interventions. Additionally, this study lays the groundwork for future adaptations of the instrument in other Spanish-speaking regions, expanding its potential impact.

Despite the robust findings, this study has some limitations. Primarily, the reliance on self-reported data may introduce subjective biases, such as social desirability or inaccurate self-perception, which can affect the precision of the executive function assessment. To strengthen the validity of the EFEES, future research should include complementary objective, performance-based measures and conduct thorough nomological validation, including concurrent and predictive validity assessments. Addressing these points will enhance the psychometric robustness and broader applicability of the scale.

## Data Availability

The raw data supporting the conclusions of this article will be made available by the authors, without undue reservation.
