# Supplementary material for: Assessing executive functioning in higher education: development and structural validation of a new self-report scale
Source: Front Psychol. 2025 Jun 26;16:1613290. doi: 10.3389/fpsyg.2025.1613290 (PMC12242968; doi:10.3389/fpsyg.2025.1613290)
Supplement: Supplementary file 1 [file Supplementary_file_1.pdf]

Front. Psychol. 16:1613290.

doi: 10.3389/fpsyg.2025.1613290

Assessing executive functioning in higher education: development and structural validation of a new self-report scale

Zamora-Lugo S, Reynoso-Alcántara V, Sanchez-Lopez J, Vergara-Lope S, Ocampo-Gómez E, García-Gomar ML, Torres-González C, Avecilla-Ramírez GN, Carbajal-Valenzuela, Calderón M, Vázquez-Moreno A, Flores-González R, Contreras-Ibáñez CC, Montero-Domínguez FÁ, Mejía-Castillo AdJ, Abundis-Gutierrez A, Sánchez-Cid JE, Osorio-Guzmán MI, García-Aguilar G, Negrete-Cortes AJ, Cisneros Báez N, Martell Ruiz LM, Campos Romero P and Cuevas-Ferrera RdF

Appendix 1. Operational definitions and behavioral indicators of the dimensions included in the instrument.

Reference authors	Definition	Subcomponents	Behavioral Indicator
**Cognitive flexibility**			
Diamond (2013); Kennedy (2017); McCloskey et al. (2008); Naglieri & Goldstein (2014); Roth et al. (2005)	Ability to detect the need to change plans, activities, strategies, thoughts or emotional states and implement changes according to the demands of the context.	Detecting the need for change	The individual identifies the need to adjust plans, activities, strategies, thoughts, or emotional states based on contextual demands when solving a problem or completing a task.
		Change: Making transitions	The individual adjusts plans, activities, strategies, thoughts, or emotional states based on contextual demands when solving a problem or completing a task.
	It implies change tolerance by allowing adaptation to new situations and effectively resuming interrupted routines.	Change tolerance: Adapting to new situations	The individual can adapt to changes in plans, activities, strategies, thoughts, or emotional states in response to contextual demands without experiencing anxiety or negative emotions.
		Change tolerance: Resuming interrupted routines	The individual can seamlessly resume interrupted routines.<br>The individual can resume interrupted routines without experiencing anxiety or negative emotional states.
	It allows for divergent thinking by combining concepts in novel ways, being creative, avoiding dichotomous thinking.	Divergent thinking: Combining concepts	The individual can integrate concepts and ideas in innovative and original ways.
		Divergent thinking: Creativity	The individual can generate innovative and creative solutions to problems.<br>The individual can think flexibly and avoid dichotomous, all-or-nothing reasoning.
	It is associated with the ability to see the perspective of the other.	Non-egocentric thinking: Seeing the perspective of the other	The individual can understand and consider others' perspectives.<br>The individual acknowledges and respects that everyone has their own way of doing things.
**Inhibitory control**			
Dawson & Guare (2010); Diamond (2013); Greenstone (2011); Kennedy (2017); McCloskey et al. (2008); Naglieri & Goldstein (2014); Roth et al. (2005)	Ability to regulate the perception of stimuli and the sequence of thoughts, feelings, and behavioral responses. This skill enables the individual to interrupt ongoing sequences or inhibit the emergence of impulsive reactions.	Stop/Interrupt	The individual can consciously halt the perception of stimuli and disrupt ongoing sequences of thoughts, feelings, and behavioral responses by considering contextual cues.
		Inhibit: Thinking before acting	The individual can pause and reflect before acting, thoughtfully evaluating contextual cues.<br>The individual can resist the impulsive urge to perceive specific stimuli and the spontaneous emergence of thoughts, feelings, and behaviors.<br>The individual can regulate impulsive behaviors.
**Working memory**			
Dawson & Guare (2010); Diamond (2013); Greenstone (2011); Kennedy (2017); McCloskey et al. (2008); Meltzer (2010); Naglieri & Goldstein (2014); Najdowski (2017); Roth et al. (2005)	Ability to hold and manipulate information in memory while completing a task.	Retention	The individual can retain all relevant information needed to perform tasks or solve problems.
		Manipulation	The individual can actively process information by relating it to prior knowledge, categorizing, and organizing it, enhancing retention in the medium or long term.
**Attentional control**			
Dawson & Guare (2010); Diamond (2013); Kennedy (2017); McCloskey et al. (2008); Naglieri & Goldstein (2014); Najdowski (2017)	Ability to determine when and where to direct attention, what to ignore, for how long, and when to shift focus.	Alert	The individual is alert and prepared to receive and process information.
		Focused attention	The individual can direct their attention to relevant stimuli.
		Selective attention	The individual can manage their attentional resources by selecting important stimuli.
		Sustained attention	The individual can sustain focus for extended periods, even in tedious or tiring situations, or amidst multiple distractions.
		Alternating attention and concentration	The individual can shift their focus of attention based on the demands of the context.

Reference authors	Definition	Subcomponents	Behavioral Indicator
		**Emotional control**	
Dawson & Guare (2010); Kennedy (2017); Meltzer (2010); Naglieri & Goldstein (2014); Najdowski (2017); Roth et al. (2005)	Ability to recognize, interpret, reframe, and regulate one's emotional states, along with their expression.	Identification/interpretation	The individual can identify and label their own emotions accurately.
		Impact of the context	The individual recognizes situations that elicit specific emotions.
		Control of attention to emotional stimuli	The individual directs and regulates attention to navigate effectively situations that evoke emotional states.
		Interpretation/reinterpretation (cognitive control of emotional response)	The individual reinterprets the valence of stimuli that evoke different emotional states.<br>The individual modulates the intensity of their emotional experiences.
		Expression of emotion.	The individual regulates the intensity of their emotional expressions.
		**Initiation to the task**	
Dawson & Guare (2010); Greenstone (2011); Kennedy (2017); McCloskey et al. (2008); Naglieri & Goldstein (2014); Roth et al. (2005)	Ability to initiate independently tasks or projects in a timely and proactive manner.	Initiation	The individual initiates tasks or projects independently.<br>The individual does not have a tendency to procrastinate.<br>The individual recognizes factors that hinder timely task initiation—such as task difficulty, lack of clarity, difficulty assessing relevance, environmental distractions, poor time management, and challenges in resisting immediate rewards—and actively works to overcome them.
		Initiative	The individual proactively initiates self-conceived projects.
		**Planning**	
Dawson & Guare (2010); Greenstone (2011); Kennedy (2017); McCloskey et al. (2008); Naglieri & Goldstein (2014); Najdowski (2017); Roth et al. (2005)	Ability to plan actions and manage resources to achieve objectives or solve problems. Note: the execution of the plan would include: initiation of the task, monitoring and evaluation (both are part of metacognition), detection of change and change needs (both of cognitive flexibility), stopping (inhibitory control) and working memory	Identification of problems or objectives	The individual identifies problems.<br>The individual sets goals.
		Identifying steps and resources to achieve goals	The individual breaks down complex problems or ambitious goals into smaller, manageable steps.<br>The individual identifies the necessary steps to achieve a goal or solve a problem.
		Anticipation of conditions or events	The individual identifies and prioritizes the most effective steps or smaller goals to successfully achieve the final objective or solve the problem efficiently.<br>The individual identifies the resources necessary to achieve the goal.
		Goal-directed persistence	The individual remains committed and continues working until the established objectives achieved are successfully.<br>The individual ensures that competing demands or interests do not hinder the achievement of their goals.<br>The individual remains committed to their goals, even when they are challenging, require multiple steps, or demand significant effort, time, or resources.<br>The individual sets goals only when they believe they are achievable and worthwhile.<br>The individual adjusts their strategies when they fail to achieve a goal or solve a problem as planned.<br>The individual seeks help when they are unable to achieve a goal or solve a problem as planned.
		**Organization**	
Dawson & Guare (2010); McCloskey et al. (2008); Meltzer (2010); Roth et al. (2005)	Ability to use routines, sequences, templates, classification criteria, etc., to establish order in the environment, materials, information, thoughts, etc.	Systematic use of tools	The individual systematically utilizes the most suitable tools—such as routines, sequences, templates, and classification criteria—to establish order in their environment, materials, information, and thoughts.<br>The individual frequently adapts tools—such as routines, sequences, templates, and classification criteria—to enhance order and organization in their environment, materials, information, and thoughts more effectively.

Reference authors	Definition	Subcomponents	Behavioral Indicator
**Time Management**			
Dawson & Guare (2010); McCloskey et al. (2008); Meltzer (2010); Najdowski (2017)	Ability to accurately estimate available time and the duration of daily activities. It also involves efficiently managing time by prioritizing tasks effectively.	Sense of time	The individual recognizes that time is a limited resource.<br>The individual estimates the time available to complete their activities efficiently and on schedule.<br>The individual precisely assesses the time needed to complete activities.<br>The individual allocates sufficient time to handle unforeseen events and complete scheduled activities without setbacks.
		Monitor the time	The individual periodically monitors their progress to ensure they remain on schedule.
		Adjustments to the time	The individual makes necessary adjustments when falling behind schedule in their activities.<br>The individual completes their activities on time.<br>The individual consistently demonstrates punctuality.
		Prioritize	The individual avoids overcommitting to tasks that cannot be completed realistically within the available time.<br>The individual allocates time to their activities based on specific criteria of importance and priority.<br>The individual prioritizes completing the most important activities first.
**Metacognition**			
McCloskey et al. (2008); Naglieri & Goldstein (2014); Roth et al. (2005)	Self-awareness: Ability to understand and recognize one's own cognitive processes.	Knowing one's own cognition	The individual is aware of their cognitive functions, strengths, abilities, and weaknesses.<br>The individual recognizes the moments and situations in which their abilities are most effective.<br>The individual understands the moments and situations in which their abilities are less effective.<br>The individual acknowledges their limited cognitive resources, such as attention and working memory.
	Self-monitoring: Ability to supervise and evaluate our own performance.	Predict	The individual anticipates the outcomes of their actions in various situations and for different actors.<br>The individual predicts the impact of their thoughts in different situations and on various actors.<br>The individual selects the strategies they believe will be most effective in solving a problem based on their predictions.
		Monitor	The individual evaluates whether the strategies used to solve a task or problem are optimal for the given context.<br>The individual recognizes the need to implement multiple strategies to solve a task or problem.<br>The individual recognizes when adjusting their strategy is necessary to solve effectively a task or problem.
		Evaluate	The individual detects mistakes made while solving a task or problem.<br>The individual identifies errors made during the process of solving a task or problem.<br>The individual accurately evaluates their performance after completing a task or solving a problem.
		Correct	The individual promptly adjusts strategies based on contextual demands when execution errors occur.

Appendix 2. Adjusted item-total correlation coefficient.

Item	Item.Total	Alpha.Without
101	.57	.76
102	.59	.75
103	.55	.75
104	.65	.75
105	.65	.75
106	.52	.75
107	.52	.75
108	.49	.75
109	.55	.75
110	.59	.75
111	.49	.75
112	.64	.75
113	.52	.76
114	.49	.76
**115**	**.30**	.76
**116**	**.32**	.76
201	.59	.75
202	.50	.75
203	.60	.75
204	.42	.76
205	.43	.76
206	.61	.75
207	.65	.75
208	.46	.76
209	.50	.75
**210**	**.25**	.76
211	.44	.76
212	.57	.75
301	.49	.75
302	.59	.75
303	.54	.75
304	.59	.75
305	.51	.76
306	.59	.75
401	.63	.75
402	.66	.75
403	.59	.75
404	.58	.75
405	.59	.75
406	.60	.75
407	.55	.75
501	.49	.76
**502**	**.38**	.76
503	.44	.76
504	.50	.75
505	.47	.75
506	.50	.75
601	.52	.76
602	.58	.75
**603**	**.20**	.76
604	.60	.75
605	.44	.75
701	.50	.76
702	.65	.75
703	.46	.76
704	.64	.76
705	.52	.76
706	.58	.75
707	.62	.75
708	.54	.76
709	.63	.75
710	.56	.75
711	.63	.75
712	.47	.76
713	.68	.75

Item	Item.Total	Alpha.Without
714	.43	.76
801	.62	.75
802	.56	.75
803	.49	.75
804	.52	.75
805	.57	.75
806	.54	.75
807	.61	.75
808	.60	.75
809	.59	.75
901	.42	.76
902	.53	.75
903	.58	.75
904	.56	.75
905	.59	.75
906	.43	.76
907	.46	.76
**908**	**.33**	.76
909	.40	.76
910	.44	.76
911	.48	.76
1001	.60	.75
1002	.56	.76
1003	.51	.76
1004	.50	.76
1005	.60	.76
1006	.56	.76
**1007**	**.21**	.76
**1008**	**.05**	.76
1101	.45	.76
1102	.47	.76
1103	.41	.76
1104	.65	.75
1105	.58	.75
1106	.60	.75
1107	.52	.76
1108	.53	.75
1109	.62	.75
1110	.52	.76
1111	.48	.76
1112	.58	.75
SUM	1.00	.98

Note: Items that did not meet the required condition (correlation coefficient lower than .40) are shown in bold.

Appendix 3. Factor loadings from the Exploratory Factor Analysis (EFA) and standardized factor loadings from the Confirmatory Factor Analysis (CFA).

	EFA				CFA			
**Item**	**F1**	**F2**	**F3**	**F4**	**F1**	**F2**	**F3**	**F4**
101	.21	.33	-.03	.17				
102	.32	.32	-.04	.10				
103	-.02	**.61**	.09	.01		.72		
104	.10	.33	.15	.22				
105	**.49**	.13	.15	-.03	.71			
106	-.09	**.44**	.30	.00		.64		
107	.20	.06	.27	.08				
108	.06	.22	.28	.03				
109	-.09	.33	.38	.08				
110	.03	.08	**.46**	.15			.68	
111	-.01	.21	**.44**	-.03			.56	
112	**.64**	.15	.13	-.20	.72			
113	**.51**	.15	.06	-.14	.58			
114	.15	.35	.15	-.06				
201	-.05	.14	**.59**	.07			.71	
202	-.09	.27	**.46**	.00			.57	
203	-.04	.11	**.45**	.24			.69	
204	.02	**.50**	-.09	.08		.57		
205	-.05	**.64**	-.05	.00		.62		
206	.00	.06	**.57**	.12			.72	
207	-.04	.25	**.61**	-.01			.75	
208	.05	**.50**	.18	-.17		.61		
209	-.12	**.48**	.32	-.06		.67		
211	-.22	**.72**	.00	.08		.61		
212	-.04	-.11	.38	**.48**				.67
301	**.47**	.01	.38	-.31	.55			
302	.35	.06	**.42**	-.15			.69	
303	.26	-.11	**.49**	-.01			.62	
304	.36	.08	.38	-.13				
305	**.57**	.14	-.03	-.11	.57			
306	**.70**	-.04	.07	-.08	.67			
401	.20	-.20	**.59**	.15			.74	
402	.06	-.07	**.62**	.20			.78	
403	.10	-.09	.36	.34				
404	.11	-.12	**.60**	.10			.67	
405	.00	-.01	**.57**	.15			.68	
406	.11	.00	**.66**	-.04			.72	
407	.26	-.03	.29	.11				
501	-.06	**.48**	.06	.13		.63		
503	-.14	.31	.36	.02				
504	.10	.35	.33	-.18				
505	-.04	**.46**	.27	-.09		.60		
506	-.12	**.69**	.22	-.15		.65		
601	-.16	-.02	.15	**.69**				.64
602	-.04	-.14	**.56**	.33			.69	
604	.17	.05	.21	.27				
605	**.43**	-.10	.16	-.01	.50			
701	**.48**	.05	.05	-.01	.57			
702	**.48**	-.13	.14	.25	.74			
703	**.07**	.24	-.13	.38				

	EFA				CFA			
**Item**	**F1**	**F2**	**F3**	**F4**	**F1**	**F2**	**F3**	**F4**
704	.18	.19	-.02	**.41**				.79
705	.09	.27	-.17	**.45**				.66
706	**.53**	-.14	.17	.09	.66			
707	**.44**	.23	-.08	.14	.68			
708	.27	.09	-.06	.34				
709	.19	-.17	.20	**.52**				.76
710	.13	-.07	.15	**.46**				.68
711	.27	-.07	.17	.39				
712	.14	.07	.00	.33				
713	**.64**	.06	.04	.04	.77			
714	.02	.12	.14	.24				
801	**.50**	.05	-.06	.22	.71			
802	**.49**	-.06	.07	.13	.67			
803	**.60**	-.13	.03	.02	.57			
804	**.51**	.12	-.05	-.01	.60			
805	**.73**	-.18	.07	-.01	.67			
806	**.57**	-.01	-.13	.15	.63			
807	**.77**	-.07	.06	-.10	.72			
808	**.57**	.12	.04	-.06	.68			
809	**.86**	-.18	.04	-.09	.71			
901	-.05	.08	.08	**.41**				.52
902	.02	-.08	.20	**.51**				.62
903	.25	-.09	.27	.26				
904	.30	-.14	.33	.15				
905	.27	-.01	.26	.19				
906	-.01	.28	-.12	.38				
907	-.16	-.14	.17	**.72**				.56
909	-.12	.05	.23	.33				
910	-.12	.12	.00	**.55**				.55
911	.05	.02	.12	.38				
1001	.22	.37	.06	.07				
1002	.31	.21	-.03	.17				
1003	.11	**.40**	.16	-.05		.62		
1004	.32	.19	-.05	.13				
1005	.30	.31	-.04	.14				
1006	.06	**.53**	.04	.06		.72		
1101	.00	**.49**	-.23	.31		.64		
1102	.04	**.60**	-.08	.01		.64		
1103	.29	.05	-.06	.19				
1104	**.49**	.22	-.08	.11	.74			
1105	**.42**	.10	-.06	.21	.66			
1106	**.74**	-.09	.11	-.11	.71			
1107	.21	.33	-.08	.16				
1108	**.44**	.07	.01	.08	.60			
1109	**.72**	-.04	.10	-.10	.73			
1110	.32	.17	-.09	.21				
1111	**.41**	.02	.02	.10	.57			
1112	**.46**	.12	-.02	.10	.67			
**Factor correlations**								
F1	1.00							
F2	.63	1.00						
F3	.63	.51	1.00					
F4	.69	.58	.52	1.00				

Note: EFA factor loadings above .4 are shown in bold, all factors in the CFA have a $p < .05$.

Front. Psychol. 16:1613290.
doi: 10.3389/fpsyg.2025.1613290

Appendix 4. List the items for each dimension and the final factor in which they are integrated.

Subcomponents	No. Order	No. by dimension	Item<br>*Example*	Final factor
			**Cognitive flexibility**	
Detecting the need for change	25	103	Puedo identificar situaciones en las que debo cambiar mis estados emocionales para ajustarme a las demandas de mi entorno.<br>*Si de repente una situación se vuelve tensa, puedes darte cuenta de que debes controlar tu enojo si quieres interactuar mejor con tus compañeros.*<br>[I can identify situations in which I need to change my emotional state to adjust to the demands of my environment.<br>*If a situation suddenly becomes tense, you may find that you need to control your anger if you want to interact better with your peers.*]	Self-control
Change: Making transitions	23	105	Puedo realizar los cambios necesarios en mis estrategias o pensamientos para ajustarme a las demandas de mi entorno.<br>*Puedes ajustar tus estrategias de lectura o tus formas de organizar la información para adecuarlas al nivel de dificultad del texto.*<br>[I can make the necessary changes in my strategies or thoughts to adjust to the demands of my environment.<br>*You can adjust your reading strategies or ways of organizing information to suit the level of difficulty of the text.*]	Organization
	3	106	Puedo realizar los cambios necesarios en mis estados emocionales para ajustarme a las demandas de mi entorno.<br>*Si una situación lo amerita, eres capaz de pasar fácilmente de un estado de exaltación o de enojo a un estado de tranquilidad.*<br>[I can make the necessary changes in my emotional states to adjust to the demands of my environment.<br>*If a situation warrants it, you can easily go from a state of exaltation or anger to a state of tranquility.*]	Self-control
Change tolerance: Resuming interrupted routines	67	110	Puedo retomar sin dificultad mis actividades cuando se interrumpe mi rutina.<br>*Te resulta sencillo volver a tu rutina escolar después de un periodo vacacional.*<br>[I can easily resume my activities when my routine is interrupted.<br>*It's easy to get back into your school routine after a vacation period.*]	Attentional and inhibitory control
	39	111	Puedo retomar sin molestias o enojos mis actividades cuando se interrumpe mi rutina.<br>*Si sucede algo inesperado, no te causa enojo faltar a un evento escolar (aun cuando tenías planeado asistir).*<br>[I can resume my activities without discomfort or anger when my routine is interrupted.<br>*If something unexpected happens, it doesn't make you angry to miss a school event (even if you were planning to attend).*]	Attentional and inhibitory control
Divergent thinking: Combining concepts	43	112	Combino de forma ingeniosa y nueva ideas y conceptos usados previamente.<br>*Puedes innovar proponiendo una forma de explicar un fenómeno tomando ideas de otros contextos.*<br>[I creatively and innovatively combine previously established ideas and concepts.<br>*You can innovate by proposing a way to explain a phenomenon by taking ideas from other contexts.*]	Organization
Divergent thinking: Creativity	24	113	Soy creativa(o) al encontrar soluciones a los problemas.<br>*Puedes adaptar herramientas para resolver nuevos problemas.*<br>[I am creative in finding solutions to problems.<br>*You can adapt tools to solve new problems.*]	Organization

Subcomponents	No. Order	No. by dimension	Item<br>*Example*	Final factor
			**Inhibitory control**	
Stop/Interrupt	56	201	Puedo ignorar voluntariamente ciertos estímulos del entorno.<br>*Intencionadamente dejas de atender los ruidos del ambiente y te enfocas en escuchar lo que dice el maestro.*<br>[I can voluntarily ignore certain stimuli in the environment.<br>*You intentionally stop paying attention to the noises in the environment and focus on listening to what the teacher is saying.*]	Attentional and inhibitory control
	9	202	Puedo ignorar voluntariamente mis pensamientos o emociones.<br>*Intencionalmente dejas de atender tus pensamientos o emociones no relacionadas con la clase y te enfocas en escuchar lo que dice el maestro.*<br>[I can willingly ignore my thoughts or emotions.<br>*You intentionally stop attending to your thoughts or emotions unrelated to the class and focus on listening to what the teacher is saying.*]	Attentional and inhibitory control
	42	203	Puedo dejar de hacer algo cuando es necesario.<br>*Puedes dejar de ver tu celular cuando requieres poner atención a tus deberes escolares.*<br>[I can stop doing something when I need to.<br>*You can stop looking at your cell phone when you need to pay attention to your schoolwork.*]	Attentional and inhibitory control
Inhibit: Thinking before acting	5	204	Pienso antes de actuar, valorando primero las características del entorno.<br>*Cuando estás en un lugar formal -un evento académico importante- piensas cómo debes moverte y actuar, evitando moverte mucho, hacer gestos exagerados, etc.*<br>[I think before acting, first assessing the characteristics of the environment.<br>*When you're in a formal place—an important academic event—you think about how you should move and act, avoiding moving around too much, making exaggerated gestures, etc.*]	Self-control
	62	205	Pienso lo que voy a decir antes de hablar, valorando primero las características del entorno.<br>*Antes de expresar tus ideas en un grupo de personas que acabas de conocer, piensas qué tono debes usar para comunicarte, qué tipo de palabras, etc.*<br>[I think about what I am going to say before speaking, first assessing the characteristics of the environment.<br>*Before you express your ideas to a group of people you've just met, you think about what tone you should use to communicate, what kind of words, etc.*]	Self-control
	33	206	Controlo a qué presto atención y qué cosas ignoro.<br>*Te enfocas en escuchar lo que dice el maestro y evitas prestar atención a los ruidos del ambiente.*<br>[I control what I pay attention to and what things I ignore.<br>*You focus on listening to what the teacher is saying and avoid paying attention to ambient noises.*]	Attentional and inhibitory control
	70	207	Controlo la atención que le presto a mis pensamientos o emociones.<br>*Te enfocas en escuchar lo que dice el maestro y evitas atender tus pensamientos o emociones no relacionadas con la clase.*<br>[I control the attention I pay to my thoughts or emotions.<br>*You focus on listening to what the teacher is saying and avoid attending to your thoughts or emotions unrelated to the class.*]	Attentional and inhibitory control
	32	208	Controlo la forma en la que me comunico, evitando responder sólo por impulso.<br>*Evitas responder automáticamente cuando alguien hace una acusación en tu contra.*<br>[I control the way I communicate, avoiding responding only on impulse.<br>*You avoid responding automatically when someone makes an accusation against you.*]	Self-control
	63	209	Controlo mis conductas, evitando actuar por impulso.<br>*Evitas revisar compulsivamente el celular cuando estás en clase.*<br>[I control my behaviors, avoiding acting on impulse.<br>*You avoid compulsively checking your cell phone when you are in class.*]	Self-control
	47	211	Evito actuar precipitadamente.<br>*Piensas antes de responder a un mensaje.*<br>[I avoid acting rashly.<br>*You think before you respond to a message.*]	Self-control
	27	212	Le doy prioridad a actividades más importantes -aunque sean menos gratificantes- que a cosas que me generan placer.<br>*Eliges hacer tus tareas antes que ver la TV o convivir con tus amigos.*<br>[I prioritize more important activities – even if they are less rewarding – than things that generate pleasure for me.<br>*You choose to do your homework rather than watch TV or hang out with your friends.*]	Time planning and management

**Subcomponents**	**No. Order**	**No. by dimension**	**Item** ***Example***	**Final factor**
			**Working memory**	
Retention	45	301	Puedo llevar a cabo eficientemente actividades que implican un alto esfuerzo mental.<br>*Puedes hacer cálculos mentalmente con varias cifras.*<br>[I can efficiently carry out activities that involve high mental effort.<br>*You can do calculations mentally with various figures.*]	Organization
	65	302	Recuerdo fácilmente la información que acabo de analizar.<br>*Recuerdas sin problemas las ideas de un texto que acabas de leer*.<br>[I easily remember the information I just analyzed.<br>*You remember without problems the ideas of a text you have just read.*]	Attentional and inhibitory control
	14	303	Puedo llevar a cabo una tarea larga (o que requiere varios pasos) sin la necesidad de consultar las indicaciones una y otra vez.<br>*Puedes completar correctamente un proyecto sin revisar varias veces las indicaciones.*<br>[I can carry out a long task (or one that requires several steps) without the need to refer to the prompts repeatedly.<br>*You can successfully complete a project without checking the prompts multiple times.*]	Attentional and inhibitory control
Manipulation	13	305	Manipulo fácilmente información nueva relacionando el contenido con información que ya conocía.<br>*Cuando estás aprendiendo términos nuevos, en tu mente los relacionas con conceptos que aprendiste antes.*<br>[I easily manipulate new information by relating the content to the information I already knew.<br>*When you're learning new terms, in your mind you relate them to concepts you learned before.*]	Organization
	15	306	Manipulo fácilmente información nueva categorizando y organizándola.<br>*Cuando estás aprendiendo un conjunto de términos nuevos, los acomodas en tu mente de acuerdo con sus características o sus significados.*<br>[I easily manipulate new information by categorizing and organizing it.<br>*When you're learning a set of new terms, you arrange them in your mind according to their characteristics or their meanings.*]	Organization

**Subcomponents**	**No. Order**	**No. by dimension**	**Item**<br>***Example***	**Final factor**
			**Attentional control**	
Alert	35	401	Permanezco alerta en mis actividades académicas cotidianas.<br>*No sueles estar somnolienta(o) durante tus clases, al realizar tus lecturas, etc.*<br>[I remain alert in my daily academic activities.<br>*You are not usually sleepy during your classes, when doing your readings, etc.*]	Attentional and inhibitory control
Focused attention	69	402	Me concentro fácilmente en mis actividades académicas.<br>*Puedes prestar atención fácilmente cuando lees.*<br>[I easily focus on my academic activities.<br>*You can easily pay attention when you read.*]	Attentional and inhibitory control
Selective attention	21	404	Evito prestar atención a cosas irrelevantes para mis actividades académicas.<br>*Puedes evitar prestar atención a tu teléfono celular cuando estudias.*<br>[I avoid paying attention to things that are irrelevant to my academic activities.<br>*You can avoid paying attention to your cell phone when you study.*]	Attentional and inhibitory control
Sustained attention	2	405	Me mantengo concentrada(o) aún en actividades largas, cansadas, aburridas, etc.<br>*Puedes mantenerte atenta(o) durante una clase larga.*<br>[I stay focused even on long, tiring, boring activities, etc.<br>*You can stay attentive during a long class.*]	Attentional and inhibitory control
Alternating attention and concentration	54	406	Me mantengo concentrada(o) aun cuando haya en el entorno cosas que me pudieran distraer.<br>*Puedes concentrarte en tus lecturas, aun cuando haya personas hablando mientras lees.*<br>[I stay focused even when there are things in the environment that could distract me.<br>*You can focus on your reading, even when people are talking while you read.*]	Attentional and inhibitory control

Subcomponents	No. Order	No. by dimension	Item<br>*Example*	Final factor
			**Emotional control**	
Identification interpretation	41	501	Identifico las emociones que siento.<br>*Sabes muy bien cuando estás triste, enojada(o), etc.*<br>[I identify the emotions I feel.<br>*You know very well when you are sad, angry, etc.*]	Self-control
Interpretation/reinterpretation (cognitive control of emotional response)	58	505	Puedo controlarme y disminuir el malestar -dolor de estómago, sudoración, calor, etc.- que me generan algunas situaciones.<br>*Cuando hablar de cierto tema te hace enojar, puedes controlarte y disminuir las sensaciones que te genera el enojo.*<br>[I can control myself and reduce the discomfort – stomach pain, sweating, heat, etc. – that some situations generate for me.<br>*When talking about a certain topic makes you angry, you can control yourself and reduce the feelings that anger generates in you.*]	Self-control
Expression of emotion.	37	506	Controlo la intensidad con la que expreso mis emociones.<br>*Eres capaz de regular tu tono de voz y tus gestos para expresar menos enojo o tristeza de lo que en realidad sientes.*<br>[I control the intensity with which I express my emotions.<br>*You can regulate your tone of voice and gestures to express less anger or sadness than you actually feel.*]	Self-control

**Subcomponents**	**No. Order**	**No. by dimension**	**Item**<br>***Example***	**Final factor**
			**Initiation to the task**	
Initiation	48	601	Inicio mis actividades de forma oportuna, sin que sea necesario que alguien me indique que debo comenzar.<br>*Inicias tus tareas sin que alguna persona, -tus padres, compañeros, etc.- te señalen que debes hacerlo.*<br>[I start my activities in a timely manner, without the need for someone to tell me that I should start.<br>*You start your tasks without someone – your parents, classmates, etc. – telling you that you should do it.*]	Time planning and management
	34	602	Evito procrastinar, es decir, evito hacer otras cosas que me impiden concluir con mis deberes a tiempo.<br>*Evitas ver la TV, jugar, limpiar la casa, etc. en lugar de hacer tus tareas.*<br>[I avoid procrastinating, that is, I avoid doing other things that prevent me from completing my duties on time.<br>*You avoid watching TV, playing games, cleaning the house, etc. instead of doing your homework.*]	Attentional and inhibitory control
Initiative	26	605	Emprendo proyectos, realizo actividades, busco nuevas cosas que hacer, sin la necesidad de que alguien más me diga que debo hacerlas.<br>*Acostumbras a estudiar temas por tu cuenta, aprender idiomas, tomar cursos adicionales, etc.*<br>[I undertake projects, I carry out activities, I look for new things to do, without the need for someone else to tell me that I should do them.<br>*You are used to studying topics on your own, learning languages, taking additional courses, etc.*]	Organization

Subcomponents	No. Order	No. by dimension	Item *Example*	Final factor
			**Planning**	
Identification of problems or objectives	1	701	Puedo identificar problemas en mi entorno académico sin la necesidad de que otras personas me los señalen.<br>*Detectas cuando dentro de tus actividades de clase debes dar soluciones, sin que te lo pida el profesor.*<br>[I can identify problems in my academic environment without the need for other people to point them out to me.<br>*You detect when within your class activities you must give solutions, without being asked by the teacher.*]	Organization
	53	702	Establezco objetivos relacionados con mi trabajo académico.<br>*Te planteas objetivos sobre las tareas que harás en una semana, o los aprendizajes y las calificaciones que obtendrás en una unidad o periodo.*<br>[I set goals related to my academic work.<br>*You set goals for the tasks you will do in a week, or the learning and grades you will get in a unit or period.*]	Organization
Identifying steps and resources to achieve goals	64	704	Puedo identificar cuando una tarea, actividad o proyecto requiere de varios pasos para poder completarse.<br>*Puedes detectar que será necesario llevar a cabo varias actividades para tener la entrega final de un proyecto.*<br>[I can identify when a task, activity, or project requires multiple steps to complete.<br>*You can detect that it will be necessary to carry out several activities to have the final delivery of a project.*]	Time planning and management
	51	705	Puedo detectar cuando, para alcanzar un objetivo, requiero alcanzar primero objetivos más pequeños.<br>*Sabes que, para pasar una materia/módulo/asignatura, debes pasar los exámenes, cumplir con las actividades, cumplir con la asistencia, etc.*<br>[I can detect when, to reach a goal, I need to reach smaller goals first.<br>*You know that, to pass a subject/module/subject, you must pass the exams, comply with the activities, comply with the attendance, etc.*]	Time planning and management
	17	706	Defino pequeños pasos para alcanzar objetivos ambiciosos o resolver problemas difíciles.<br>*Puedes establecer las estrategias que utilizarás en cada unidad/módulo/tema para obtener buenas notas y así lograr un buen promedio.*<br>[I define small steps to achieve ambitious goals or solve difficult problems.<br>*You can set the strategies that you will use in each unit/module/topic to get good grades and thus achieve a good average.*]	Organization
Anticipation of conditions or events	20	707	Reflexiono sobre los pasos más efectivos que deberé llevar a cabo para poder alcanzar un objetivo ambicioso, antes de iniciar.<br>*Reflexionas sobre lo que debes hacer antes de iniciar con una actividad práctica, pensando en los pasos que implica, los productos que obtendrás de esta, etc.*<br>[I reflect on the most effective steps that I will have to take to be able to achieve an ambitious goal, before starting.<br>*You reflect on what you should do before starting a practical activity, thinking about the steps involved, the products you will get from it, etc.*]	Organization
Goal-directed persistence	49	709	Trabajo hasta que alcanzo los objetivos que me he establecido.<br>*Te comprometes a estudiar para acreditar tus materias con buenas calificaciones y haces lo necesario para lograrlo, hasta tener resultados positivos.*<br>[I work until I reach the goals I have set for myself.<br>*You commit to studying to accredit your subjects with good grades and you do what is necessary to achieve it, until you have positive results.*]	Time planning and management
	11	710	Hago todo lo necesario para alcanzar mis objetivos, a pesar de toparme con cosas agradables/interesantes que pudieran impedírmelo.<br>*Si te propones sacar buenas calificaciones, puedes evitar ir a una fiesta y mejor quedarte a estudiar.*<br>[I do whatever it takes to achieve my goals, despite bumping into nice/interesting things that might be holding me back.<br>*If you intend to get good grades, you can avoid going to a party and better stay to study.*]	Time planning and management
	44	713	Reflexiono sobre lo que estoy haciendo para lograr mis objetivos identificando éxitos y fracasos y la posibilidad de cambiar mis estrategias.<br>*Valoras si tus estrategias de estudio son efectivas para aprender en cierta materia, y generas alternativas en caso de que no estés teniendo éxito.*<br>[I reflect on what I am doing to achieve my goals by identifying successes and failures and the possibility of changing my strategies.<br>*You assess whether your study strategies are effective for learning in a certain subject, and you generate alternatives in case you are not succeeding.*]	Organization

Subcomponents	No. Order	No. by dimension	Item<br>*Example*	Final factor
			**Organization**	
Systematic use of tools	52	801	Utilizo formas/herramientas que me permiten organizar eficientemente mi entorno de trabajo.<br>*La organización de tu espacio de trabajo te permite acceder fácilmente a todo lo que necesitas para llevar a cabo tus actividades.*<br>[I use forms/tools that allow me to efficiently organize my work environment.<br>*Organizing your workspace allows you to easily access everything you need to carry out your activities.*]	Organization
	57	802	Utilizo formas/herramientas que me permiten organizar mis materiales de trabajo eficientemente.<br>*Tienes un sistema para organizar tus libros, cuadernos, documentos de lectura, etc., que te permite encontrar algún material cuando lo necesitas.*<br>[I use shapes/tools that allow me to organize my work materials efficiently.<br>*You have a system for organizing your books, notebooks, reading documents, etc., which allows you to find some material when you need it.*]	Organization
	4	803	Utilizo formas/herramientas que me permiten organizar mis ideas a la hora de escribir textos académicos.<br>*Usas plantillas que te ayudan a colocar la información completa y en orden al escribir un ensayo, proyecto, etc.*<br>[I use forms/tools that allow me to organize my ideas when writing academic texts.<br>*You use templates that help you put the information complete and in order when writing an essay, project, etc.*]	Organization
	31	804	Utilizo formas/herramientas que me permiten organizar eficientemente mis pensamientos al hablar.<br>*Haces un listado de las cosas que debes decir, piensas en el orden en el que debes expresar las ideas, piensas previamente en ejemplos o argumentos, etc.*<br>[I use forms/tools that allow me to efficiently organize my thoughts when speaking.<br>*You make a list of the things you should say, you think about the order in which you should express the ideas, you think beforehand about examples or arguments, etc.*]	Organization
	10	805	Utilizo formas/herramientas que me permiten organizar y clasificar eficientemente información.<br>*Tienes un sistema para clasificar conceptos y vincularlos con otros términos, y así, recordar más fácilmente su significado.*<br>[I use forms/tools that allow me to efficiently organize and classify information.<br>*You have a system for classifying concepts and linking them to other terms, so you can more easily remember their meaning.*]	Organization
	12	806	Genero nuevas formas/herramientas que me permiten organizar mejor mi entorno de trabajo y mis materiales.<br>*Sueles cambiar la organización de tu espacio de trabajo para hacer todo más accesible.*<br>[I generate new forms/tools that allow me to better organize my work environment and materials.<br>*You often change the organization of your workspace to make everything more accessible.*]	Organization
	46	807	Genero nuevas formas/herramientas que me permiten organizar mejor mis ideas a la hora de escribir textos académicos.<br>*Adaptas plantillas para mejorar el orden de la información al escribir un ensayo, proyecto, etc.*<br>[I generate new forms/tools that allow me to better organize my ideas when writing academic texts.<br>*You adapt templates to improve the order of information when writing an essay, project, etc.*]	Organization
	28	808	Genero formas/herramientas que me permiten organizar mejor mis pensamientos al hablar.<br>*Adaptas nuevas estrategias para preparar un discurso que tienes que hacer en público.*<br>[I generate forms/tools that allow me to better organize my thoughts when speaking.<br>*You adapt new strategies to prepare a speech that you must make in public.*]	Organization
	18	809	Genero formas/herramientas que me permiten organizar y clasificar mejor información.<br>*Adaptas sistemas de clasificación, plantillas, esquemas, etc., para clasificar mejor los conceptos y vincularlos con otros términos, y así, recordar más fácilmente su significado.*<br>[I generate forms/tools that allow me to better organize and classify information.<br>*You adapt classification systems, templates, schemes, etc., to better classify concepts and link them with other terms, and thus, remember their meaning more easily.*]	Organization

Subcomponents	No. Order	No. by dimension	Item<br>*Example*	Final factor
			**Time Management**	
Sense of time	30	901	Tengo presente que dispongo de una cantidad de tiempo limitada al día.<br>*Tienes claro que en el día debes dedicar tiempo para asearte, alimentarte, descansar y realizar tus actividades escolares, etc.*<br>[I am aware that I have a limited amount of time per day.<br>*You are clear that during the day you should dedicate time to wash yourself, eat, rest and carry out your school activities, etc.*]	Time planning and management
	7	902	Tengo claro el tiempo del que dispongo para realizar, de forma oportuna, mis actividades académicas.<br>*Sabes muy bien que sólo cuentas con X número de horas para realizar tus tareas entre semana.*<br>['I am aware of how much time I have to complete my academic activities on time.<br>*You know very well that you only have X number of hours to do your tasks during the week.*]	Time planning and management
Adjustments to the time	68	907	Entrego mis actividades puntualmente.<br>*Entregas tus tareas a tiempo.*<br>[I deliver my activities on time.<br>*You turn in your assignments on time.*]	Time planning and management
Prioritize	29	910	Dedico más tiempo a las actividades que considero más importantes o difíciles.<br>*Das prioridad a las tareas que tienen mayor peso en tu calificación.*<br>[I spend more time on the activities that I consider more important or difficult.<br>*You prioritize the tasks that have the most weight in your grade.*]	Time planning and management

**Subcomponents**	**No. Order**	**No. by dimension**	**Item** *Example*	**Final factor**
			**Metacognition**	
			Awareness	
Knowing one's own cognition	8	1003	Soy consciente de mis debilidades y fortalezas para regular mis emociones.<br>*Sabes qué tan eficiente eres para comprender lo que sientes, regular como expresas tus emociones, etc.*<br>[I am aware of my weaknesses and strengths to regulate my emotions.<br>*You know how efficient you are at understanding what you feel, regulating how you express your emotions, etc.*]	Self-control
	61	1006	Conozco las situaciones en las que se me facilita/dificulta comprender y regular mis emociones.<br>*Sabes cuales son las situaciones en las que te resulta más fácil comprender lo que sientes, regular como expresas tus emociones, etc.*<br>[I know the situations in which it is easier or harder for me to understand and regulate my emotions.<br>*You know which are the situations in which it is easiest for you to understand what you feel, regulate how you express your emotions, etc.*]	Self-control
			Self-monitoring	
Predict	55	1101	Preveo las consecuencias de mis acciones ante diversas situaciones.<br>*Piensas en qué sucederá si faltas a clases, o si repruebas un examen.*<br>[I foresee the consequences of my actions in various situations.<br>*You think about what will happen if you miss class, or if you fail an exam.*]	Self-control
	59	1102	Preveo el impacto que tendrán mis acciones en las personas que me rodean.<br>*Te detienes a pensar en qué consecuencias tendrá ser grosero con un compañero.*<br>[I foresee the impact my actions will have on the people around me.<br>*You stop to think about what consequences being rude to a partner will have.*]	Self-control
	60	1104	Reflexiono sobre la utilidad de mis estrategias para resolver problemas mientras las llevo a cabo.<br>*Te detienes a pensar si lo que estás haciendo te permitirá concluir el proyecto que estás realizando para una materia/módulo/asignatura.*<br>[I reflect on the usefulness of my problem-solving strategies as I carry them out.<br>*You stop to think if what you are doing will allow you to conclude the project you are doing for a subject/module/subject.*]	Organization
Monitor	50	1105	Cuando resuelvo un problema, me detengo a verificar si requiero algunas estrategias adicionales o diferentes para resolverlo.<br>*Te detienes a pensar si leer el libro de texto es suficiente para comprender un problema de estadística, o, además, debes ver un video explicativo.*<br>[When I solve a problem, I stop to check if I require some additional or different strategies to solve it.<br>*You stop to think about whether reading the textbook is enough to understand a statistical problem, or, in addition, you should watch an explanatory video.*]	Organization
	36	1106	Reflexiono sobre la utilidad de las estrategias que uso para analizar/organizar información mientras estudio.<br>*Te detienes a pensar si elaborar cuadros sinópticos, hacer un resumen, elaborar un cuestionario, etc., te está permitiendo aprender.*<br>[I reflect on the usefulness of the strategies I use to analyze/organize information while studying.<br>*You stop to think if drawing up synoptic tables, making a summary, preparing a questionnaire, etc., is allowing you to learn.*]	Organization
Evaluate	16	1108	Acostumbro a dedicar tiempo para evaluar si las estrategias que utilicé fueron efectivas para finalizar una tarea o resolver un problema.<br>*Al concluir un proyecto reflexionas sobre lo que hiciste bien y lo que hiciste mal.*<br>[I usually spend time evaluating whether the strategies I used were effective in completing a task or solving a problem.<br>*At the end of a project, you reflect on what you did right and what you did wrong.*]	Organization
	38	1109	Reflexiono sobre la utilidad de las estrategias para analizar y organizar información que empleé para estudiar los contenidos académicos.<br>*Piensas en qué tanto te fue de utilidad hacer un cuadro comparativo, un mapa conceptual, etc.*<br>[I reflect on the usefulness of the strategies for analyzing and organizing information that I used to study academic content.<br>*You think about how useful it was for you to make a comparative chart, a concept map, etc.*]	Organization
	6	1111	Reflexiono sobre mi desempeño al concluir una actividad académica.<br>*Dedicas tiempo para pensar en lo que te faltó estudiar, o lo que no te quedo claro después de un examen.*<br>[I reflect on my performance at the end of an academic activity.<br>*You take time to think about what you missed studying, or what was not clear to you after an exam.*]	Organization
Correct	66	1112	Reflexiono sobre la necesidad de cambiar de estrategia al resolver un problema o completar una tarea, tomando en cuenta los errores que estoy cometiendo y los cambios en el contexto.<br>*Mientras resuelves un examen, piensas constantemente en tus errores y cómo corregirlos.*	Organization

	[I reflect on the need to change my strategy when solving a problem or completing a task, considering the mistakes I am making and the changes in the context. *While solving a test, you are constantly thinking about your mistakes and how to correct them.*]

**Subcomponents**	**No. Order**	**No. by dimension**	**Item**<br>***Example***	**Final factor**
			**Verification Items**	
	22	1201	Conozco y recuerdo mi nombre.<br>*Puedes decir cómo te llamas.*<br>[I know and remember my name.<br>*You can tell your name.*]	Verification Item
	19	1202	Sé leer textos en español.<br>*Puedes leer el título de un libro.*<br>[I know how to read texts in Spanish.<br>*You can read the title of a book.*]	Verification Item
	40	1203	Soy un estudiante inscrito en el bachillerato.<br>*Te encuentras cursando algún semestre/periodo/grado de bachillerato.*<br>[I am a student enrolled in high school.<br>*You are studying a semester/period/bachelor's degree.*]	Verification Item
